# Quantitative Analysis of Trade Position Shifts of China and the United States in the Indian Ocean Rim Trade Networks Using a Weighted Centrality Approach

**DOI:** 10.3390/e27030262

**Published:** 2025-03-01

**Authors:** Lihua Yuan, Changqing Song, Xiaoqiang Chen, Manjun Zhang, Menghan Yang

**Affiliations:** 1 State Key Laboratory of Earth Surface Processes and Resource Ecology, Beijing Normal University, Beijing 100875, China; geobnuer@mail.bnu.edu.cn; 2 School of Remote Sensing and Information Engineering, North China Institute of Aerospace Engineering, Langfang 065000, China; 3 Center for Geodata and Analysis, Faculty of Geographical Science, Beijing Normal University, Beijing 100875, China; 4 Faculty of Geography, Tianjin Normal University, Tianjin 300387, China

**Keywords:** trade network, complex network analysis, the Indian Ocean Rim (IOR), United States, China, weighted centrality indicators, Fisher optimal segmentation method

## Abstract

The Indian Ocean Rim (IOR) is a crucial hub for global commerce, possessing key maritime corridors and competitive markets for China and the United States. Assessing the evolving positions of China and the United States in regional trade provides critical insights into their economic competition. This study quantitatively investigated their changing positions in the IOR trade networks from 1992 to 2020 through an interdisciplinary approach combining the Fisher optimal segmentation, chord-diagram visualization, and five weighted centrality indicators, including two advanced metrics derived from physical current flow theory. The results reveal a significant shift in their trade positions in the IOR trade networks across four phases (1992–2002, 2003–2008, 2009–2014, and 2015–2020); in particular, the United States occupied a dominant position in the IOR trade networks until 2008, after which China rose to the central trading position, as reflected in its top ranking across four weighted indicators (excluding weighted authority centrality). In machinery and transport equipment (SITC7), China also surpassed the United States in 2008 and further consolidated its supremacy, driven by its strong manufacturing capabilities and the growing demand from the IOR countries. Meanwhile, the United States experienced a noticeable decline but maintained substantial influence as a key importer. This research develops a quantitative framework that integrates the temporal segmentation with weighted centrality indicators to provide insights into the dynamics and structural changes of trade networks across sectors and regions.

## 1. Introduction

### 1.1. Background

The Indian Ocean Rim (IOR) hosts vital maritime corridors connecting Asia, Africa, and Europe, and it has abundant and diverse natural resources and a vast population [1,2]. As a pivotal region for global commodity transportation and energy supply, it is vital to the global economy, accounting for 30% of global trade [3,4,5]. This strategic and economic importance has positioned the IOR as a key area of international focus and great power competition, particularly between China and the United States [1,6,7,8]. China has increased its economic significance in this region through infrastructure investments and strengthened trade ties across the energy, mineral, and manufacturing sectors. In contrast, the United States—the dominant player in this region since the end of the Cold War—has leveraged its technological and high-end manufacturing trade to maintain its economic influence and counterbalance China’s growing economic presence.

This competition is deeply intertwined with the trade relations of the IOR countries, with both economies seeking to secure access to critical markets and resources [1,6]. As these trade relations provide a measurable reflection of the evolving balance of power between China and the United States in this region, quantitatively assessing their evolving positions within the IOR trade network is crucial for understanding the manifestation of their competition and economic strategies.

### 1.2. Literature Review

#### 1.2.1. Progress in International Trade Network Research

Complex network analysis, as a powerful tool to study the relations and structure of a system composed of nodes and links, has been extensively applied in the international trade research [9,10,11,12]. It allows trade flows between countries to be modeled as an interdependent network, representing countries as nodes and their trade flows as links. A large number of trade network studies have emerged by using network visualization [13,14,15,16], overall network indicators [14,15], network structure methods [4,14,16,17,18,19], and node centrality methods [10,11,12,13,14,17,18] to investigate the features and structure of trade networks, as well as the countries’ positions within global and regional trade networks.

Previous research focused on global trade networks [10,11,12,13,14,15,16,17,18] and highly integrated regions, such as Europe [20], Southeast Asia [21,22], and the BRI region [19,22,23], to provide valuable insights into trade structures and the evolution of countries’ roles or positions within these networks. However, there remains a notable gap in the systematic investigation of trade networks within the IOR as an emerging region. Specifically, the trade positions and competitive dynamics of the two major external powers in this region, namely, China and the United States, have not been thoroughly revealed using quantitative methods. Addressing this gap is essential for understanding international trade networks.

#### 1.2.2. Application of Centrality Measures in Trade Network Analysis

Centrality measures in complex network analysis are practical tools for quantitatively assessing the importance or position of countries within trade networks [10,12,23,24,25,26,27,28]. Degree, closeness [29], betweenness [30], and eigenvectors [31] are widely employed unweighted centrality measures used in global and regional trade networks to capture different aspects of countries’ importance [12,22,27,28]. However, these unweighted indicators only consider nodes’ connections, failing to account for the weights of links (i.e., volume of trade flows between countries). Consequently, they cannot accurately capture the importance of countries within trade networks. To overcome this limitation, weighted centrality indicators allow for incorporating the weights of links (i.e., trade flow magnitudes) into network analyses, thus providing more accurate tools to quantify a country’s importance and roles in global or regional trade networks. For instance, weighted degree centrality (strength centrality) [28] and weighted eigenvector centrality [32] were easily extended by incorporating trade flow weights, enabling the accurate measurement of node positions in trade networks. However, the other key centrality measures—namely, betweenness and closeness—assume that information transfer follows the shortest paths and lack a suitable transformation for calculating trade flow weights [33].

Recent advancements, such as current flow betweenness and current flow closeness centralities, derived from the current flow model in physics, provide feasible and effective alternatives [24,25,26,27,28,29,30,31,32,33,34,35,36]. These two indicators extend traditional measures by simulating the propagation of current flow across all possible paths between nodes in a network, with higher probabilities assigned to trade flows of greater magnitude. This feature makes them particularly suitable for weighted networks and provides a more accurate representation of countries’ positions within complex trade systems.

#### 1.2.3. Research on IOR Trade

The IOR represents a highly complex trade network shaped by intra-regional economic agreements and strong external linkages [4,5,8], resulting in a multi-layered structure characterized by regional and global interactions. This complexity is further reflected in the IOR’s heavy reliance on major external powers, including China, the United States, and Japan. However, scholarly attention on the IOR has primarily focused on geopolitical and economic issues [37,38], particularly descriptive analyses of strategic initiatives by China and the United States. Consequently, there is a lack of systematic and quantitative evaluations focused on the evolving trade positions of the United States and China within the IOR trade networks, thus hindering a comprehensive understanding of the shift of the two powers’ trade positions and their economic competition in this region.

### 1.3. Research Gaps and Contributions

To the best of our knowledge, there have been no studies focused on systematic quantitative analyses of the dynamic evolution of the trade positions of China and the United States in the IOR trade networks, especially using weighted centrality indicators. This study aimed to address these gap by integrating the Fisher optimal segmentation method, chord diagram visualization, the current flow model, and complex network analysis to construct a weighted centrality indicator framework for quantitative analysis of the changing positions of China and the United States in the IOR trade networks. This framework can more accurately assess a country’s different aspects and roles in global or regional trade networks, and this study provided insights into the competitive dynamics between the two big powers in the IOR trade networks.

The remainder of this article is structured as follows: Section 2 introduces the study area, trade flow data, the segmentation method for time series data, and five weighted centrality indicators. Section 3 analyzes the characteristics of China and the United States’ trade with the IOR countries across four phases, as well as the evolution of their positions in the IOR trade networks and machinery and transport equipment (SITC7) trade networks in particular. Finally, Section 4 discusses the results, while Section 5 concludes this paper and outlines future research directions.

## 2. Data and Methods

### 2.1. Study Area

**Figure 1:**
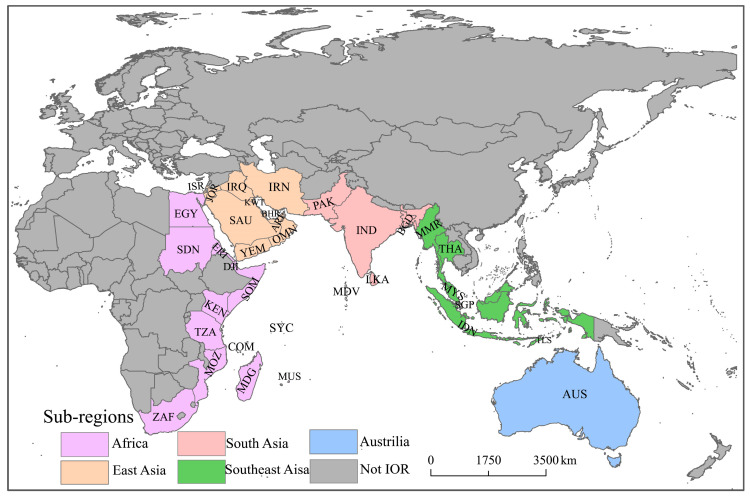
The geographical distribution of countries in the Indian Ocean Rim (IOR).

The IOR is a strategically vital maritime corridor connecting the Pacific and Atlantic Oceans and is situated between the eastern coasts of Africa, Asia, and Australia. Academically, the IOR is defined in broad and narrow terms [1,2]. The narrow definition focuses on the 36 coastal countries bordering the Indian Ocean [5]. This study adopted the narrow definition due to its emphasis on maritime transport and trade. Under this narrow definition, the IOR includes Australia in Oceania, six Southeast Asian nations, five South Asian nations, 11 West Asian nations, and 13 African nations (Figure 1). These countries play crucial roles in the context of maritime transportation, international trade, and geoeconomic activities.

This region exhibits economic diversity. Developed economies, such as Australia and Singapore, have per capita GDPs exceeding USD 50,000, while emerging markets—such as India, Indonesia, and Saudi Arabia—present rapid economic growth. In contrast, the underdeveloped East African nations face significant challenges but hold the potential for future development. Such economic diversity not only shapes the structure and dynamics of the IOR trade networks but also fosters opportunities for economic cooperation between trading partners [3,4,5,39].

### 2.2. Source of Trade Flow Data

This study applied international merchandise trade flow data for 1992–2020, obtained from the Atlas of Economic Complexity (AEC) developed by Harvard University. Due to its broad coverage of approximately 250 countries and regions worldwide and its reliability [40], the AEC dataset is a vital and widely used trade flow data for trade-related studies [4,8,22,41,42].

Obtained from original trade flow data from the UN Comtrade Database, the AEC dataset has been refined by cross-referencing countries’ reported exports and imports data [43] to improve the accuracy and reliability of bilateral trade flows. Compared with the UN Comtrade data, the AEC dataset provides more accurate and consistent trade data, particularly for countries whose trade data in the UN Comtrade Database are often incomplete, such as Iraq, Somalia, Yemen, and African nations in the IOR. Therefore, the AEC dataset is well-suited for exploring the IOR trade networks. 

### 2.3. Methods

#### 2.3.1. Fisher Optimal Segmentation Method to Partition Time Series Trade Flow Data

Considering the significant changes in global and regional economic and political dynamics from 1992 to 2020, the trade patterns within the IOR have likely experienced significant structural changes. Therefore, this study segmented the time series data of trade flows between China, the United States, and the IOR countries to capture their evolution scientifically.

Traditional methods for temporal segmentation, such as historical-event-based approaches or uniform interval divisions (e.g., five- or ten-year intervals), often fail to capture meaningful differences between successive stages. The time series constrained cluster method, such as stratigraphically constrained cluster, uses agglomerative hierarchical clustering (a local optimization solution) to identify breakpoints, and thus, does not guarantee globally optimal results [44,45]. These constraints can lead to inaccurate breakpoints, which do not guarantee the capture of the underlying shifts in time series data, potentially distorting the interpretation of temporal dynamics.

To address these above shortcomings, this study adopted the Fisher optimal segmentation method, a globally optimal and statistically robust approach designed to segment time series data by minimizing the variance within clusters and maximizing the differences between them [46]. Unlike traditional segmentation methods, the Fisher method is data-driven and objective, allowing for the identification of breakpoints based on the inherent patterns of the data rather than on traditional subjective segmentation approaches. This method provides a more accurate and reliable approach for capturing structural shifts in time-series data, such as the significant changes in China’s spatial economy and regional industrial shifts [47], as well as global political structures [48] over different periods. By adopting Fisher optimal segmentation, we ensure that the identified breakpoints reflect genuine transitions in the underlying data. This offers a clearer understanding of the evolving positions of China and the United States. The principles and computational steps of the Fisher method are as follows:

Step 1: Definition of the diameter of a cluster (one segment). The diameter of a cluster of time series data is defined as the sum of the squared deviations between all sample data within the cluster and its averaged value. The formula used is as follows:

(1) $$D(i,j)=\sum_{t=i}^{j}{\left(X_{t}-{\overset{-}{X}}_{ij}\right)}^{\prime }(X_{t}-{\overset{-}{X}}_{ij}),$$

where ${\overset{-}{X}}_{ij}=\frac{1}{j-i+1}\sum_{t=i}^{j}X_{t}$.

Step 2: Definition of the loss function. The loss function *e*[*b*(*n*,*k*)], which measures the total intra-cluster distances for a partition *b*(*n*,*k*), is defined as

(2) $$e[b(n,k)]=\sum_{t=i}^{k}D(i_{t},i_{t+1}-1)$$

Therefore, the goal of Fisher optimal segmentation is to identify a partition *b*(*n*,*k*) that minimizes the above loss function; that is, to minimize *e*[*b*(*n*,*k*)].

Step 3: Recursive calculation of the loss function. Fisher proposed a recursive method based on the loss function to recursively calculate and determine the position of each optimal breakpoint for a given number of segments *k*, as follows:

(3) $$e[b(n,k)]=\underset{k\leq j\leq n}{\min}\{e[b(j-1,k-1)]+D(j,n)\}$$

Step 4: Determination of the optimal number of segments. As Fisher optimal segmentation does not inherently determine the optimal number of segments due to its focus on error minimization within a given segment number, a commonly used method to estimate the optimal number of segments is the elbow method, which plots the curve of the minimum error function (*e*) against *k*. The optimal value of *k* is typically selected at the “elbow” point of the curve, where the rate of decrease in *e* significantly slows, indicating a trade-off between minimizing the error and maintaining model simplicity.

Step 5: Determination of breakpoints and output of the optimal partition. After computing the minimal loss when *k* is determined as the optimal number of partitions, the optimal breakpoints are determined as follows: The breakpoint that minimizes *e*[*b*(*n*,*k*)] = *e*[*b*(*j_k_* − 1, *k* − 1)] + *D*(*j_k_*,*n*) is identified to obtain the *k*-th segment. Then, the above process is iteratively repeated to determine all the segments. In this way, the optimal segmentation of the time series data is obtained.

#### 2.3.2. Methodology for Construction of Weighted Directed IOR Trade Networks

To quantitatively examine the changing positions of China and the United States, this study constructed weighted directed IOR trade networks, where each link incorporated a trade flow direction and its magnitude. For each directed link from Country A to Country B, the weight was determined by A’s exports to B. Similarly, the weighted link from B to A was weighted by B’s exports to A. Japan, as the third-largest external trading partner of the IOR, was also incorporated into the IOR trade networks as a key node in order to ensure a rigorous analysis.

Network chord diagrams were used to visualize the IOR trade networks by excluding bilateral trade flows between China, the United States, and Japan, which emphasized their trade connections with the IOR countries rather than the high trade volumes between the three countries. However, when calculating the weighted centrality indicators, the bilateral trade flows between the three countries were incorporated to avoid underestimating their positions in the IOR trade networks.

By adopting this methodology, the chord diagrams of IOR trade networks depict the trade relationships between China, the United States, and IOR countries, while the weighted centrality indicators quantify their importance in the regional trade networks.

#### 2.3.3. Five Weighted Centrality Indicators to Assess the Positions of China and the United States in the IOR Trade Networks

In complex network analysis, centrality measures are used to evaluate the importance of nodes quantitatively. As introduced in Section 1.2.2, weighted centrality measures provide a more accurate tool to quantify the importance of nodes in a network by incorporating the magnitudes of links into the calculation. Therefore, this study introduced five weighted centrality measures: weighted eigenvector centrality, weighted hub centrality, weighted authority centrality, and current flow closeness and current flow betweenness centralities, with the latter two based on the physical current flow model. These measures were used to assess the different aspects of a country’s importance in a weighted directed trade network.

Weighted eigenvector, hub, and authority centrality measures [32] can be easily obtained by replacing the connections in their unweighted formulas with weights. However, for unweighted betweenness and closeness centralities, there is a lack of a suitable transformation for calculating such weights. To address this limitation, we introduced two advanced weighted indicators for the IOR trade network analysis; namely, current flow betweenness and current flow closeness centrality, which integrated current flow theory from physics with complex network analysis [34,35,36]. Unlike traditional unweighted betweenness and closeness centralities, which assume that information flows along the shortest path, these two current flow centralities model information as randomly moving particles within the network, which can be compared with the decision-making process of individuals with bounded rationality. Thus, this approach provides a more rational and realistic quantification, making them particularly suitable for capturing the complexities of weighted international trade networks. Overall, these five weighted centrality indicators provide a multi-dimensional perspective and a comprehensive framework to quantitatively assess the positions of China and the United States in the IOR trade networks.

(1) Current flow betweenness centrality: Based on Kirchhoff’s current law in physics, the current flow betweenness centrality measures the average amount of current flowing through a specific node in a network, quantifying its importance in facilitating resource flows. In the global trade network, where various elements (e.g., goods and information) are exchanged between countries, this indicator assesses a country’s role as a bridge or intermediary in a trade network by evaluating the probability of resources passing through it along random paths. The formula used is as follows:

(4) $$\mathrm{CFBC}(v_{i})=\frac{2}{\left(n-1)(n-2\right)}\sum_{j\neq k}^{n}I_{i}(v_{s},v_{t})$$

where *I_i_*(*v_s_*,*v_t_*) represents the current flow through node *v_i_* as the current moves from node *v_s_* to node *v_t_*.

(2) Current flow closeness centrality: This is defined as the reciprocal of the average potential difference between all node pairs passing through the node. A trade network measures a country’s overall proximity to all other countries in the network by simulating the random flow of resources and highlighting countries that act as central nodes in connecting different traders within trade networks. The formula is given by

(5) $$\mathrm{CFCC}(v_{i})=\frac{n-1}{\sum_{j\neq i}Ø(v_{i},v_{j})}$$

where Ø(*v_i_*,*v_j_*) represents the potential difference between nodes *v_i_* and *v_j_*, corresponding to the effective resistance.

(3) Weighted eigenvector centrality: Eigenvector centrality measures a node’s influence within a network by considering its direct connections and the importance of its connected nodes. The weighted version incorporates connection weights and can better evaluate a node’s influence more precisely, particularly in the context of international trade networks [48]. The weighted eigenvector centrality is defined as follows:

(6) $$\mathrm{WEC}(v_{i})=\frac{1}{\lambda }\sum_{j}^{n}W(v_{i},v_{j})\mathrm{WEC}(v_{j})$$

where *λ* is an eigenvalue, *W*(*v_i_*, *v_j_*) depicts the weight between *v_i_* and *v_j_* (i.e., the trade flow strength from country *v_i_* to country *v_j_*), and *n* represents the number of nodes connected to *v_i_* (i.e., the number of trading partners for country *v_i_*).

(4) Weighted hub centrality: The weighted hub centrality depends on the weighted authority scores of the nodes a given node is linked to, highlighting its role as a producer or distributor in a directed network. In a weighted directed trade network, this measures a node’s importance as an exporter or source of connections to key importers. The weighted hub score is calculated as follows:

(7) $$\mathrm{WHC}(v_{i})=\alpha \sum_{j=1}^{n}\mathrm{WE}(v_{i},v_{j})\mathrm{WAC}(v_{j})$$

where *WE*(*v_i_*, *v_j_*) represents the weight of the link from node *v_i_* to node *v_j_* (e.g., the trade flow from exporter *v_j_* to importer *v_i_*); *WAC*(*v_j_*) is the authority score of node *v_j_*; and *α* is a normalization constant, which is used to ensure that the scores converge during the iterative computation.

(5) Weighted authority centrality: The weighted authority is influenced by the weighted hub scores of the nodes linking to it, and it is used to measure a node’s importance as an import destination (or consumption market) in a trade network. The weighted authority score is calculated as follows:

(8) $$\mathrm{WAC}(v_{i})=\beta \sum_{j=1}^{n}\mathrm{WI}(v_{i},v_{j})\mathrm{WHC}(v_{j})$$

where *WI*(*v_i_*, *v_j_*) represents the weight of the link from node *v_j_* to node *v_i_* (e.g., the trade flow from exporter *v_j_* to importer *v_i_*), and *β* is a normalization constant, which is used to ensure that the scores converge during the iterative computation. These two formulas form a recursive relationship and can be calculated using methods from linear algebra (e.g., eigenvalue decomposition), yielding the weighted hub and authority scores for each node.

## 3. Results

### 3.1. Four-Phase Analysis of Trade Between China and the United States with the IOR Countries Using the Fisher Segmentation Method

**Figure 2:**
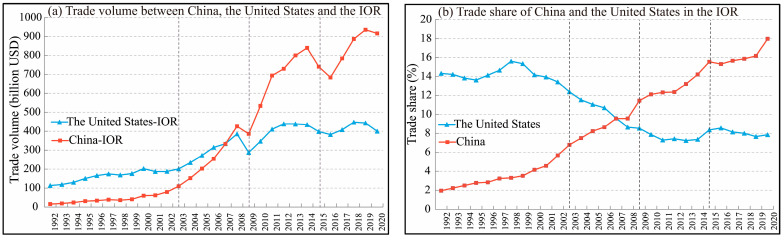
Trade volumes and shares of China and the United States with the IOR from 1992 to 2020.

To scientifically analyze the evolution of the trade positions of China and the United States within the IOR trade networks, the Fisher segmentation method—selected for its capacity to detect structural shifts in trade patterns through variance minimization—was applied to divide their trade flow values into four distinct phases (Figure 2): the first (1992–2002), second (2003–2008), third (2009–2014), and fourth phases (2015–2020). These phases basically aligned with critical events, reflecting not only quantitative changes in the trade flows but also qualitative shifts in the global economic dynamics and trade interactions between China, the United States, and the IOR countries.

The first phase (1992–2002), identified by the Fisher segmentation method, saw post-Cold War economic globalization drove most IOR countries to adopt liberalization policies. As a result, the United States emerged as the dominant trading partner, with an average share of 14%, while China, which had not yet fully integrated into regional trade, ranked ninth, with a share of less than 4%, reflecting its peripheral role.

In the second phase (2003–2008), the Fisher segmentation method identified 2003 as the statistical breakpoint—a lagged reflection of policy impacts from China’s designation as an Indian Ocean Rim Association for Regional Cooperation Dialogue Partner in 2000 and its accession to the World Trade Organization (WTO) in 2001. These policy shifts, e.g., delayed tariff reduction schedules and gradual supply chain reorientation, propelled China’s rise as a regional trade competitor. By 2008, China’s trade share rose to 8.8%, surpassing the United States’ share of 8.65% (Figure 2), marking its transition from a peripheral role to a core trader.

In the third phase (2009–2014), China’s trade volume with the IOR continued to grow rapidly, with its average trade share rising to 13%. Meanwhile, the United States prioritized domestic economic recovery and its trade share continued to drop, with an average value of 7.5%. This divergence solidified China’s trade importance as a pivotal trading partner, making a clear shift in economic influence in the IOR.

China’s average trade share rose to 16%, more than double that of the United States (7%) in the fourth phase (2015–2020). This growth was bolstered by the Belt and Road Initiative (BRI), which strengthened China’s trade with the IOR countries through expanded economic cooperation. Key mechanisms included reducing the trade costs via transport infrastructure projects, which facilitated trade expansion, attracted foreign investment, and contributed to improve the people’s standard of living in this region [49]. In contrast, the United States’ trade presence in the IOR continued to decline.

The analysis demonstrates China’s rise from a marginal economic participant to a pivotal trading force within the IOR from 1992 to 2020, while the United States experienced a decline in trade importance. These shifts reflect the evolving global priorities and China’s deepening integration across IOR economies.

### 3.2. Evolution of Positions of China and the United States in the IOR Trade Networks over the Four Phases

**Figure 3:**
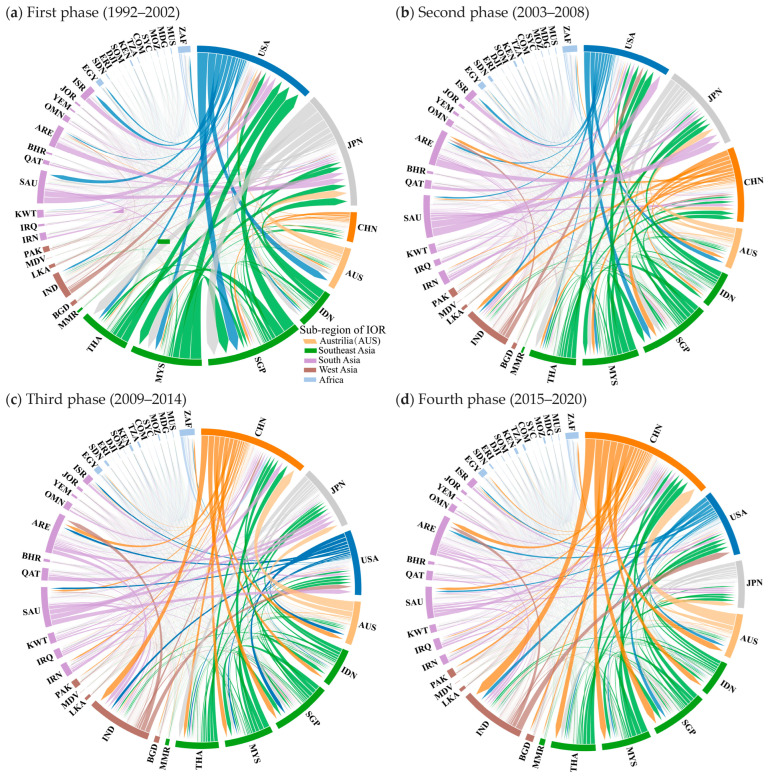
Chord diagram visualization of the IOR trade networks over the four phases (1992–2020). Note: the thickness of a line represents the volume of trade flow, while the size of a node represents the total trade volume of a country with other countries within a trade network.

**Figure 4:**
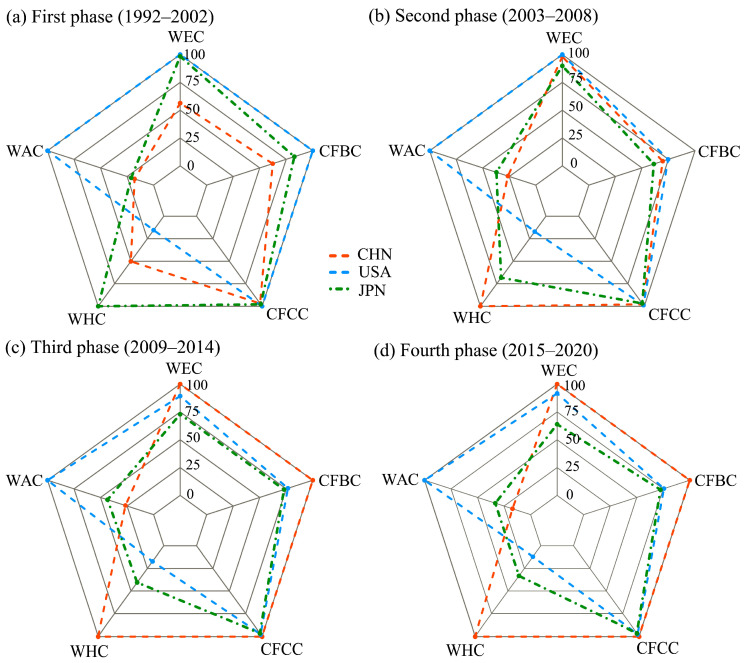
Radar chart visualization of the five weighted centrality indicators for China and the United States over the four phases (1992–2020). Note: CFBC and CFCC denote the current flow betweenness and closeness centralities, respectively, while WEC, WAC, and WHC refer to the weighted eigenvector, authority, and hub centralities, respectively.

This section uses the chord diagrams depicted in Figure 3 and the five weighted centrality indicators visualized in Figure 4 to provide a visual and quantitative analysis of the evolution of China and the United States’ trade positions in the IOR trade networks over four distinct phases.

#### 3.2.1. Evolution of the Trade Relations Between China, the United States, and the IOR Countries over the Four Phases: Chord Diagram Visualization

Chord diagrams clearly and intuitively represent how the trade linkages evolved between China, the United States, and the IOR countries (Figure 3). The node sizes in the diagrams correspond to each country’s total trade volume, while the thickness of each chord represents the intensity of the trade flow. Across the four phases, the chord diagrams highlight China’s growing trade engagement with the IOR countries, contrasted by a relative decline in the trade presence of the United States in this region.

In the first phase, the United States dominated the IOR trade network, characterized by the largest node and thick chords connecting to its major trade partners in the IOR, including Southeast Asian countries (Thailand, Malaysia, and Singapore), West Asian economies (Saudi Arabia and Israel), and Australia. These strong trade links highlighted the United States’ prominent position in the region’s trade. Meanwhile, China’s node size was much smaller and it presented much thinner trade connections with the IOR countries, reflecting its marginal position in the IOR trade networks.

In the second phase, China’s trade links with the IOR countries expanded both in breadth and intensity, as reflected in the chord diagram in Figure 3b. The larger node size and thicker chords signified a surge in trade flows with the IOR countries, particularly those in Southeast and West Asia. Although the United States still had the largest node size, the gap between China, the United States, and Japan had narrowed significantly, highlighting China’s significantly growing influence and signaling the beginning of a shift in their trade positions within the IOR.

In the third phase, a significant shift in the IOR trade network could be observed, where China became the top trade partner of the IOR countries. The chord diagram in Figure 3c vividly depicts China’s strong trade connections, particularly with Southeast Asian nations (e.g., Singapore and Malaysia) and West Asian economies, strengthening its position as the top trade partner. Meanwhile, the United States continued to decline in node size and chord thickness compared with China, illustrating its diminishing position within the IOR trade networks.

In the fourth phase, China’s trade relations with the IOR countries grew significantly more extensive and robust, as reflected in China’s larger node and more intensive chords in the trade network in Figure 3d when compared to the previous phase shown in Figure 3c. In contrast, while the United States continued to experience a decline, with its node further shrinking, it sustained more significant trade relations with India. The chord diagram clearly illustrates China’s expanding trade presence across the IOR and the United States’ declining importance within the IOR during this phase.

These chord diagrams illustrate the evolving trade relationships between China, the United States, and the IOR countries, visually underscoring China’s growing trade integration with the IOR countries and the concurrent relative decline of United States’ trade engagement.

#### 3.2.2. Weighted-Centrality-Based Quantitative Analysis of Trade Position Shifts of China and the United States in the IOR Network Across the Four Phases

The five weighted centrality indicators—current flow betweenness, current flow closeness, weighted eigenvector, weighted hub, and weighted authority centralities—provide a quantitative framework for analyzing the evolving positions of China and the United States in the IOR trade networks. By examining the values of the five normalized weighted centrality indicators, which range from 0 to 100 (Figure 4), the dynamic trajectories of trade positions for the two countries over the four phases can be quantitatively measured and tracked.

Current flow betweenness centrality captures a country’s role as a facilitator between two countries that are not directly connected within the network [10]. In the first phase, the United States had a perfect score of 100, indicating its dominant role as a trade intermediary, whereas China’s centrality was 62.23, reflecting a more peripheral role. During the second phase, China’s centrality increased to 69.60, surpassing Japan’s 61.22 and narrowing the gap with the United States, whose centrality declined to 74.72—signaling China’s growing influence. In the third phase, China surpassed the United States to claim the top position with a perfect score of 100, emphasizing its crucial role as a key trade connector in the IOR. In the fourth phase, China retained its leadership position.

Current flow closeness centrality measures how closely a country is connected to all other countries within a trade network. In the first phase, the United States scored 100, showcasing its superior efficiency in trade exchange, while China scored 96.93—lower than Japan’s 97.68. In the second phase, China’s closeness centrality improved to 97.87, surpassing Japan’s 96.86 and nearing that of the United States, which remained at 100. In the third phase, China achieved a score of 100, exceeding the United States (97.71) and highlighting its improvement in effectively facilitating trade. In the fourth phase, China maintained its top position, while the United States scored 97.68.

Weighted eigenvector centrality quantifies a country’s general influence within a trade network. In the first phase, the United States held the dominant position with a perfect score of 100, reaffirming its strong and well-established connectivity across the regional market. In contrast, China, with a score of 56.36, occupied a relatively peripheral position. However, by the second phase, China’s score surged to 98.10, which surpassed Japan (89.83) and closely approached the United States (100), marking its rapid rise in regional trade significance. In the third phase, China overtook the United States with a perfect score of 100, while the United States experienced a further decline. In the fourth phase, China maintained its score of 100, consistently outperforming both the United States (91.62) and Japan (63.89), thereby solidifying its position as the most influential trader in the IOR trade network.

The eigenvector centrality can be decomposed based on the direction of trade flows to assess a country’s authority centrality as a market (i.e., receiving imports from key traders) or hub centrality as a producer and exporter (i.e., sending exports to major markets).

In terms of weighted hub centrality, in the first phase, China’s score of 50.15 was higher than the United States’ 15.48 but lagged behind Japan’s score of 100. By the second phase, China achieved a score of 100, where it increasingly improved its position as an important exporter. This dominance persisted throughout the third and fourth phases, with China maintaining its top score of 100, while the United States experienced a significant decline to 11.97.

For weighted authority centrality, throughout the four phases, the United States consistently held the top position with a score of 100, underscoring its robust import demand and dominant role as a central player in global trade. In contrast, China’s weighted authority centrality exhibited a dynamic trajectory, where it initially experienced growth but later declined. By the fourth phase, China’s score decreased to 17.08—returning to a level comparable with that observed in the first phase, signaling a potential loss of influence as a key importer or connector to major exporting nations.

The analysis of the five centrality indicators reveals divergent trajectories for China and the United States within the IOR trade network. China evolved from a peripheral participant to a dominant force, excelling in the current flow betweenness, current flow closeness, weighted eigenvector centrality, and weighted hub centrality. By the third phase, China had overtaken the United States in multiple key centralities, solidifying its position as the primary trade intermediary and hub in the IOR. In contrast, the United States retained its dominance regarding weighted authority centrality, reflecting its strong import demand; however, its relative decline in other measures signals a reduced overall position in the IOR trade network when compared with China.

### 3.3. Evolution of the Positions of China and the United States in the Machinery and Transport Equipment (SITC7) Trade Networks in the IOR

Classified as SITC7 in the UN Standard International Trade Classification (SITC), the machinery and transport equipment sector—encompassing essential industrial items, including machinery, vehicles, and associated equipment—is crucial for the worldwide economy. Based on our calculations, the proportion of SITC7 imports and exports in the overall trade volumes between China, the United States, and the IOR countries varied between 30% and 50%, reflecting its critical importance in the trade between them. Thus, this section analyzes the changing positions of China and the United States in the SITC7 trade networks within the IOR.

#### 3.3.1. The Evolution of Trade Relations Between China, the United States, and the IOR Countries in the SITC7 Trade Network: Chord Diagram Visualization

**Figure 5:**
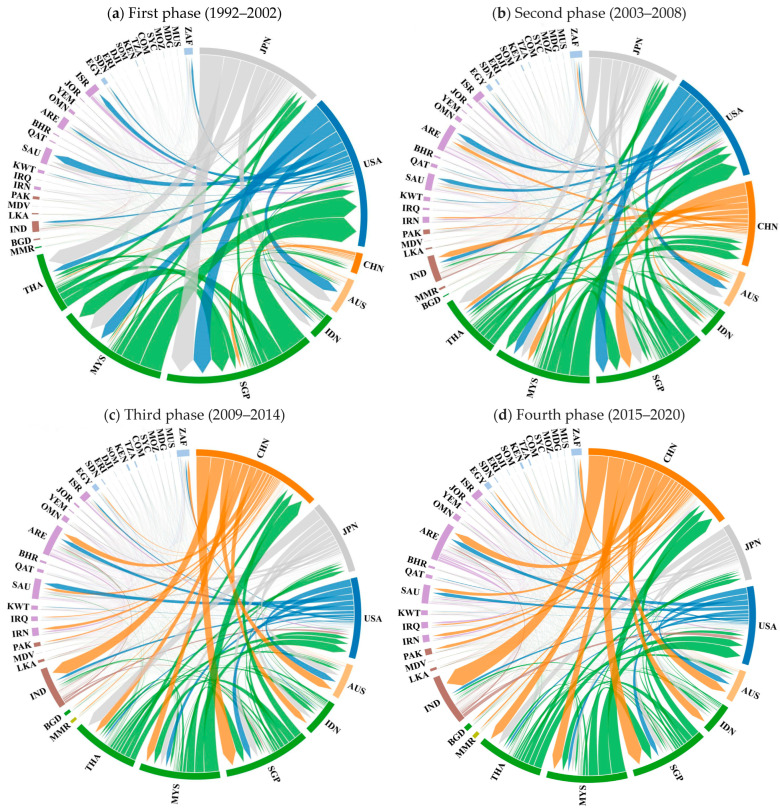
Chord diagram visualization of machinery and transport equipment (SITC7) trade networks in the IOR over the four phases (1992–2020).

The chord diagrams provided in Figure 5 clearly depict the evolving trade relations between China, the United States, and the IOR countries in the SITC7 sector over the four periods.

During the first phase, the United States dominated the IOR SITC7 trade network through its advanced manufacturing capabilities and robust domestic demand, and thus, maintained strong trade ties with major partners in Southeast Asian nations and key Western Asian countries, including Saudi Arabia and the UAE. Japan emerged as the second-largest node by fostering export-driven partnerships, particularly with Southeast Asian nations. In stark contrast, China only occupied a peripheral trade position characterized by thinner trade links with the IOR countries.

In the second phase, China experienced substantial SITC7 trade flow expansion across IOR countries, notably intensifying exchanges with Southeast Asian nations. This growth not only strengthened China’s presence in this region but also initiated competition with the United States in terms of the SITC7 exports in the IOR markets.

The third phase marked a structural shift in the IOR SITC7 trade network, where China surpassed both Japan and the United States to become the top trading partner with the IOR. In this period, China notably increased its SITC7 exports to Australia, Southeast Asian countries, India, the UAE, Saudi Arabia, and Iran, while it maintained strong import channels from Singapore, Malaysia, and Thailand (Figure 5c). In contrast, Japan and the United States exhibited diminished influence in the IOR SITC7 trade network, as evidenced by their reduced node sizes and thinner trade flows, relative to the representations in the second phase in Figure 5b.

In the fourth phase, China consolidated its dominant position as the leading node in the IOR SITC7 trade network, through which it established extensive trade connections across the region. This demonstrates that China’s production outputs met the demands of of regional markets for SITC 7 products, which were critical to the industrialization needs of most IOR countries. In contrast, Japan’s importance in the SITC7 trade network diminished, while the United States maintained its significance.

#### 3.3.2. Quantitative Analysis of Trade Position Shifts of China and the United States in the IOR SITC7 Trade Networks Using Weighted Centrality Indicators

**Figure 6:**
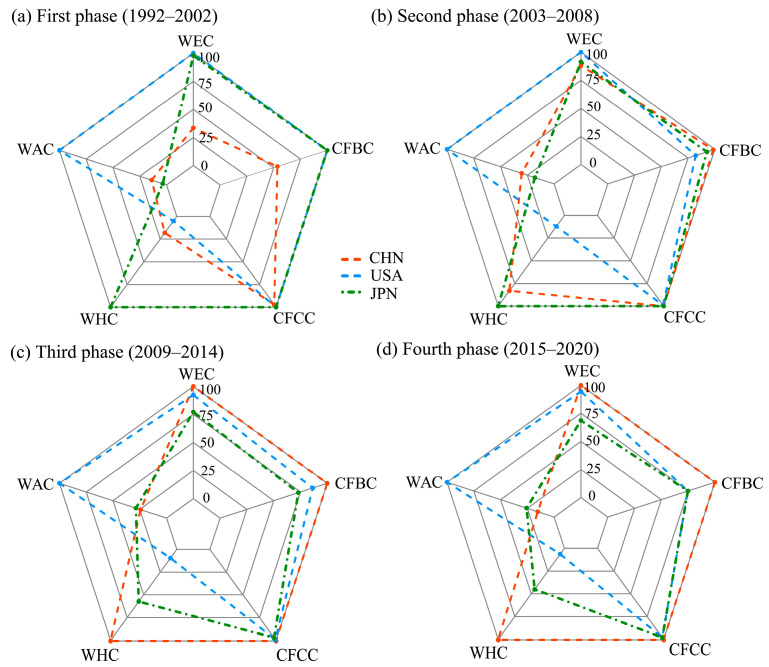
Radar chart visualization of the five weighted centrality indicators for China and the United States in the IOR SITC7 trade networks over the four phases (1992–2020). Note: CFBC and CFCC denote the current flow betweenness and closeness, respectively; WEC, WAC, and WHC refer to the weighted eigenvector, authority, and hub centralities, respectively.

This section applies the same five weighted centrality indicators to analyze how the positions of China and the United States evolved in the SITC7 sector. All indicators were normalized to the same (0–100) scale (Figure 6).

In the first phase, the current flow betweenness centrality of both the United States and Japan scored 100, which highlighted their robust roles as “pass-through” centers, whereas China lagged with a score of 53.47. However, China’s betweenness centrality surged to 98.54 in the second phase, whereas Japan’s score declined to 74.81, signaling a relative weakening of its intermediary role. During the third phase, China surpassed both the United States and Japan, where they reached a score of 100. In the fourth phase, China retained its top position with a score of 100, which solidified China’s role as the leading trade connector in the IOR SITC7 trade network, while the United States fell to 75.65.

In the first phase, both the United States and Japan achieved a current flow closeness centrality score of 100, demonstrating their superior efficiency in trade connectivity with other countries, while China was slightly behind at 97.21. In the second phase, China’s current flow closeness centrality increased to 99.99, surpassing both the United States and Japan. China achieved a perfect score of 100 in the third phase, and maintained this position through the fourth phase. Meanwhile, the United States slightly declined to 97.68.

The weighted eigenvector centrality of the United States scored 100 in the first phase, followed by Japan (at 97.83), while China (with a score of 33.53) was only one-third that of the United States. China rapidly increased its score to 88.13 in the second phase, where China significantly narrowed the gap with the United States (100), while Japan slightly declined (to 91.37). In the third phase, China surpassed the United States with a perfect score of 100 and maintained this position in the fourth phase, while the United States’ centrality continued to decline, which highlighted China’s growing dominance in the IOR SITC7 trade network.

Japan achieved a perfect weighted hub centrality score of 100 in the first phase, and thus, established itself as the most critical node in the IOR SITC7 network. In comparison, China scored half of Japan’s score, placing it in relatively peripheral position, although it ranked higher than the United States (which scored 15.48). During the second phase, China rapidly achieved a perfect score of 100, solidifying itself as the central producer and exporter in the SITC7 industry—a position it maintained throughout the third and fourth phases. From the third phase onward, Japan experienced a significant decline, reflecting its diminishing role as a key producer and exporter hub in the SITC7 sector.

The United States dominated import-driven trade flows in the SITC7 industry, achieving a perfect score of 100 in the first phase, while China (17.94) and Japan (3.60) lagged significantly behind. Over the next three phases, the United States maintained its leading position with a consistent score of 100. In contrast, China made modest progress in the second (26.28) and third phases (26.57), but declined to 17.08 in the fourth phase, underscoring the persistent gap in the SITC7 import demand between China and the United States.

The quantitative analysis revealed that China surpassed the United States in four weighted indicators, including the current flow betweenness, current flow closeness, eigenvector, and hub centralities, by the third phase. However, the United States retained its superiority in the weighted authority centrality, especially due to its strong import demand and connections to important exporters, even as its influence in the other four aspects declined. Overall, China transitioned from a peripheral role to become the central trader in the IOR SITC7 trade network, contrasting with the United States’ residual import leverage.

## 4. Discussion

### 4.1. A General Framework for Analyzing the Evolution of a Country’s Position in a Weighted Directed Trade Network: Integration of Fisher Optimal Segmentation, Visualization, and Weighted Centralities

In view of the complexity of international trade networks and the heterogeneous magnitudes of trade flows, this study utilized a comprehensive framework aimed at understanding the evolution of a country’s position in a weighted directed trade network, thus capturing the dynamics of trade relations. This framework integrated three methods: first, the Fisher optimal segmentation technique was utilized to segment the time series data of trade flows into different phases; second, network chord diagrams were used to illustrate the geographic distribution and relative strength of trade flows; third, five weighted centrality indicators—namely, current flow betweenness and closeness centralities, along with weighted eigenvector, hub, and authority centralities—were employed to quantitatively assess the magnitude of the different aspects roles of countries participating in weighted directed trade networks. A comparative analysis of this framework with previous studies is provided below.

The proposed integrated framework was utilized to explore the evolution of the positions of China and the United States in the IOR trade networks during the period 1992–2020. Based on this framework, the approximately 30 years (1992–2020) were divided into four distinct phases using the Fisher optimal segmentation method, and distinct temporal patterns were identified. Chord diagrams for the four phases revealed clear patterns in the geographic distribution and relative strengths of trade flows, particularly between China, the United States, and the IOR countries, which highlighted China’s increasing integration into the economies of this region. The five weighted centrality measures quantitatively illustrated the progressive strengthening of China’s position within the IOR trade networks. Conversely, the United States experienced a relative decline in most weighted centrality indicators, except for the weighted authority centrality.

Importantly, the centrality indicators not only quantified countries’ influence [50] but also served as proxies for systemic vulnerability. An analysis of the Dieselgate scandal revealed that environmental violations by a central corporate actor (Volkswagen) triggered contagion effects across the German automotive industry—a phenomenon that highlights how a high connectivity in networked systems can amplify systemic risks [51]. By analogy, in global trade networks, the high centrality score of a nation like China may similarly accelerate the transmission of economic shocks through interconnected trade channels. The observed centrality shifts imply that China’s growing network prominence may increase its capacity to transmit economic shocks through weighted trade linkages (e.g., bilateral trade volumes) to structurally aligned economies, such as Southeast Asian nations with high trade volumes with China. The proposed integrated framework can also offer insights into understanding systemic vulnerabilities in the IOR’s trade.

### 4.2. Comparison with Existing Studies

Compared with previous studies on trade networks, which widely use unweighted centrality measures, this study provided a more comprehensive and dynamic framework for analyzing trade networks’ dynamics. For instance, the studies [10,11,12,13,14,16,23] employed measures of unweighted centrality (such as closeness, betweenness, and eigenvectors) to evaluate the importance of major participants in the global and regional trade networks, but both failed to capture the heterogeneous nature of magnitudes in trade flows. In contrast, this study introduced five weighted centrality indicators, especially current flow betweenness and closeness centralities developed from the current flow model from physics. These indicators can be compared to decision-making processes of individuals under limited rationality [48], offering a more accurate evaluation of countries’ intermediary roles and the efficiency of trade connectivity. Additionally, weighted hub, authority, and eigenvector centralities provide a quantitative assessment of traders’ different facets of influence (export, import and general influence) in a realistic trade network. Thus, through the use of these weighted indicators by incorporating magnitudes in trade flows, this study provided a more accurate and comprehensive quantitative assessment of the positions of countries within the trade network.

Additionally, the application of the Fisher optimal segmentation method with a global optimal solution enabled the scientific identification of temporal phases in the time series data of trade flows. This method is an advanced method compared to subjective approaches—such as historical event-based or uniform interval division (e.g., five- or ten-year intervals), which are commonly used in previous studies on global trade [13,14,15,16,17] and regional trade [24,28] trade networks. It also outperforms the quantitative segmentation method based on local optimal solutions—that involves stratigraphically constrained clustering, which were recently introduced to regional trade research [8,23,52].

Using this framework, this study corroborated previous findings regarding the IOR trade patterns [5,8], particularly the strengthening of China’s trade position and the declining presence of the United States. However, we also provide insights by quantitatively revealing how China’s position evolved from a peripheral role to a central trader in this region, in contrast to the United States’ decline, through the application of network chord diagrams and five weighted centrality indicators.

### 4.3. Driving Factors of the Shift in the Trade Positions of the United States and China in the IOR Trade Networks from 1992 to 2020

The empirical results indicate notable shifts in the trade stances of China and the United States within the IOR networks from 1992 to 2020. This section analyzes the macroeconomic and policy-based factors that drove these changes.

China’s increasing trade position in the IOR was driven by its expanding manufacturing capacity, which has generated substantial demand for energy and raw materials from the IOR, as well as by its economic cooperation measures. China’s policy developments—including its dialogue partnership with the Indian Ocean Rim Association for Regional Cooperation (2000), WTO accession (2001), the China–ASEAN Free Trade Zone (2010), and the Belt and Road Initiative (2013)—have progressively intensified economic ties with IOR nations through the maintenance of good political relations, promotion of greater economic cooperation, reduction of trade barriers, transport projects, and fostering of investment opportunities. As a result, China’s trade share in the IOR increased from 2% in 1992 to 16% in 2020, which strengthened its trade position in the IOR. In contrast, the United States’ role in the IOR weakened, declining its trade share from 14% in 1992 to 7% in 2020. This shift was closely linked to reduced energy dependence on Western Asian countries since 2010 and diminished policy engagement with Southeast Asia. Consequently, trade relations with these two regions have declined in comparison to China, as shown in the chord diagrams.

China’s improved trade position in the IOR was also influenced by the peer effect, whereby countries or groups adopt similar behaviors by observing and imitating the successful practices of others [53,54]. This effect facilitated bilateral import–export activities between China and the IOR countries. Specifically, the peer effect among IOR nations—which was driven by emulating the successful strategies of neighboring countries in technology adoption and market expansion with China—contributed to advancing industrial cooperation and policy coordination between China and the IOR states, helping to increase China’s trade position in the IOR.

China and the United States have established differentiated functional positions in the IOR trade landscape. China has solidified its position as a principal trading partner through sustained export competitiveness, which functioned as the primary supplier of medium-technology manufactured goods for numerous IOR economies. Conversely, although the United States’ post-Cold War hegemonic dominance in the IOR trade weakened, it maintains structural leverage as a critical export market for IOR nations. These trade patterns, visualized through chord diagrams, revealed asymmetric interdependencies, with China reinforcing its supply-side significance and the U.S. maintaining demand-side predominance in regional trade dynamics.

## 5. Conclusions

The IOR is a vital global marketplace for trade competition between China and the United States. Due to the complexity and heterogeneity of trade relations between China, the United States, and the IOR countries, this study conducted an in-depth and quantitative analysis to systematically trace the evolution of their positions. Specifically, this study developed a qualitative framework integrating the Fisher optimal segmentation method, chord diagram visualization, the current flow model from physics, and complex network analysis to construct a weighted centrality indicator system. By segmenting the IOR trade flow data into four phases (1992–2002, 2003–2008, 2009–2014, and 2015–2020), this framework provided insight into the evolution of the positions of China and the United States within IOR trade networks.

This study revealed a significant shift in the core trade positions of two major external actors within the IOR trade networks from 1992 to 2020. The United States maintained a dominant role in IOR networks until 2008. However, following this period, China rose as a prominent player, boosted by strong export growth, the development of strengthened economic cooperation with the IOR countries, and the implementation of the BRI.. This transformation was supported by four centrality indicators (excluding weighted authority centrality). Furthermore, China surpassed the United States to become the leading trader in the pivotal sector of machinery and transport equipment. This shift highlights China’s increasing economic influence and the United States’ declining presence in the IOR trade networks.

This study analyzed the shifting positions of China and the United States in the IOR trade networks and explored the underlying drivers of these changes. It introduced a new approach to studying complex trade networks by integrating temporal segmentation techniques with five weighted network centrality indicators. In the future, this analysis will be expanded in two key directions. First, it will investigate the IOR’s trade networks across three critical sectors—energy commodities (e.g., crude oil), labor-intensive goods (e.g., textiles), and technology-intensive industries (e.g., transportation equipment)—to uncover sectoral competition patterns between China and the United States in the IOR. Second, the study will integrate value-added data from the Eora MRIO database (1992–2015), which covers most IOR countries. The study will then use trade-flow data from 2015 to 2020 to extend the value-added series to 2020. This combined dataset will then be used to analyze China’s and the United States’ positions in the IOR value chain networks to uncover each country’s captured value at different stages of production. By combining cross-sector analysis with value chain analysis, this dual approach will provide a more comprehensive assessment of the dynamics of trade competition between China and the United States in this region.

## Data Availability

The trade flow data are available at https://dataverse.harvard.edu/dataverse/atlas (accessed on 18 March 2022).

## References

[1] Bouchard C., Crumplin W. (2010). Neglected No Longer: The Indian Ocean at the Forefront of World Geopolitics and Global Geostrategy. J. Indian Ocean Rim.

[2] Feng C.L. (2019). Geo-environmental and Geo-risk of the Indian Ocean Region from the Perspective of “21st Century Maritime Silk Road”. Indian Ocean Econ. Pol. Rev..

[3] Rensburg S.J.J.V., Viviers W., Cameron M., Parry A. (2018). Identifying Export Opportunities Between IORA Member States Using the TRADE-DSM Methodology: A Case Study Involving South Africa and Thailand. J. Indian Ocean Reg..

[4] Yuan L., Chen X., Song C., Cao D., Yi H. (2021). Spatiotemporal Evolution and Determinant Factors of the Intraregional Trade Community Structure of the Indian Ocean Region. ISPRS Int. J. Geo Inf..

[5] Liang S.L., Guo T. (2021). The Analysis of the Structural Characteristics of the Manufacturing Trade Network Between China and the Indian Ocean Rim. Int. Bus..

[6] Butt K.M., Siddiqui S.J. (2021). Growing Chinese Presence in the Indian Ocean: Prospects and Challenges. J. Strateg. Stud..

[7] Fatima Q., Jamshed A. (2015). The Political and Economic Significance of Indian Ocean: An Analysis. S. Asian Stud..

[8] Yuan L., Chen X., Song C., Cheng C., Shen S. (2021). Spatiotemporal Patterns of Geoeconomics of the Countries in the Indian Ocean Region. Acta Geogr. Sin..

[9] Herman P.R. (2022). Modeling Complex Network Patterns in International Trade. Rev. World Econ..

[10] Antonietti R., Falbo P., Fontini F., Grassi R., Rizzini G. (2022). The World Trade Network: Country Centrality and the COVID-19 Pandemic. Appl. Netw. Sci..

[11] Du R., Wang Y., Dong G., Tian L., Liu Y., Wang M., Fang G. (2017). A Complex Network Perspective on Interrelations and Evolution Features of International Oil Trade, 2002–2013. Appl. Energy.

[12] Fan Y., Ren S., Cai H., Cui X. (2014). The State Role and Position in International Trade: A Complex Network Perspective. Econ. Modell..

[13] De Benedictis L., Tajoli L. (2011). The World Trade Network. World Econ..

[14] Liu Z., Wang T., Chen W. (2019). The Rise of China and Change of the Global Trade Network During 1980–2018. Prog. Geogr..

[15] Zhou M., Wu G., Xu H. (2016). Structure and Formation of Top Networks in International Trade, 2001–2010. Soc. Netw..

[16] Chen W., Wang N. (2022). Visualizing the Changing Geographies of International Trade, 2000–19. Reg. Stud. Reg. Sci..

[17] Cepeda-López F., Gamboa-Estrada F., León C., Rincón-Castro H. (2018). The Evolution of World Trade from 1995 to 2014: A Network Approach. J. Int. Trade Econ. Dev..

[18] Coquidé C., Lages J., Ermann L., Shepelyansky D.L. (2022). COVID-19 Impact on International Trade. Entropy.

[19] Fu L., Ma X., Dou Z., Bai Y., Zhao X. (2024). Key Node Identification Method Based on Multilayer Neighbor Node Gravity and Information Entropy. Entropy.

[20] Chen W., Zhang H., Tang Z., Yu Z. (2023). Assessing the Structural Connectivity of International Trade Networks Along the “Belt and Road”. PLoS ONE.

[21] Krings G.M., Carpantier J.-F., Delvenne J.-C. (2014). Trade Integration and Trade Imbalances in the European Union: A Network Perspective. PLoS ONE.

[22] Chen X., Yuan L., Song C. (2023). Investigating Merchandise Trade Structure in the RCEP Region from the Perspective of Regional Integration. J. Geogr. Sci..

[23] Chen X., Yuan L., Song C. (2022). Comparison on the Trade Development and Influence of China and the U.S. in the Surrounding Areas of China. Geogr. Res..

[24] Song Z., Che S., Yang Y. (2018). The Trade Network of the Belt and Road Initiative and Its Topological Relationship to the Global Trade Network. J. Geogr. Sci..

[25] Chong Z.H., Qin C.L., Pan S. (2019). The Evolution of the Belt and Road Trade Network and Its Determinant Factors. Emerg. Mark. Financ. Trade.

[26] Zhang H., Liu W., Liu Z. (2021). Trade Patterns and Its Influencing Factors Between China and Important Node Regions Under “the Belt and Road” Initiative: A Case Study of Transcaucasia Countries. Geogr. Geo-Inf. Sci..

[27] Freeman L.C. (1979). Centrality in Social Networks: Conceptual Clarification. Soc. Netw..

[28] Iapadre P.L., Tajoli L. (2014). Emerging Countries and Trade Regionalization: A Network Analysis. J. Policy Model..

[29] De Benedictis L., Nenci S., Santoni G., Tajoli L., Vicarelli C. (2014). Network Analysis of World Trade Using the BACI-CEPII Dataset. Glob. Econ. J..

[30] Brandes U. (2008). On Variants of Shortest-Path Betweenness Centrality and Their Generic Computation. Soc. Netw..

[31] Bonacich P. (1987). Power and Centrality: A Family of Measures. Am. J. Sociol..

[32] Barrat A., Barthélemy M., Pastor-Satorras R., Vespignani A. (2004). The Architecture of Complex Weighted Networks. Proc. Natl. Acad. Sci. USA.

[33] Sajedianfard N., Hadian E., Samadi A.H., Dehghan Shabani Z., Sarkar S., Robinson P.A. (2021). Quantitative Analysis of Trade Networks: Data and Robustness. Appl. Netw. Sci..

[34] Reyes J., Schiavo S., Fagiolo G. (2010). Using Complex Network Analysis to Assess the Evolution of International Economic Integration: The Cases of East Asia and Latin America. J. Int. Trade Econ. Dev..

[35] Brandes U., Fleischer D. (2005). Centrality Measures Based on Current Flow. Annual Symposium on Theoretical Aspects of Computer Science.

[36] Newman M.E.J. (2005). A Measure of Betweenness Centrality Based on Random Walks. Soc. Netw..

[37] Yang Y.S. (2019). The Economic Rationale Behind the Concept of “Indo-Pacific”. Glob. Rev..

[38] Feng F., Ma D. (2024). LDA-Based Identification and Evolutionary Analysis of Hot Topics in Indo-Pacific Strategy. Inf. Res.

[39] Cao D., Chen X., Song C., Yuan L., Du B. (2022). A Comprehensive Assessment and Spatiotemporal Evolution Analysis of National Risk of China in the Indian Ocean Region. Sci. Geogr. Sin..

[40] Hausmann R., Hidalgo C.A. (2011). The Network Structure of Economic Output. J. Econ. Growth.

[41] Ballard-Rosa C., Carnegie A., Gaikwad N. (2018). Economic Crises and Trade Policy Competition. Br. J. Polit. Sci..

[42] Yue J., Zhou S. (2018). Democracy Comparative Advantage: Evidence from Aggregated Trade Data, 1962–2010. World Dev..

[43] Bustos S., Yıldırım M.A. (2022). Production Ability and Economic Growth. Resour. Policy.

[44] Grimm E.C. (1987). CONISS: A FORTRAN 77 Program for Stratigraphically Constrained Cluster Analysis by the Method of Incremental Sum of Squares. Comput. Geosci..

[45] Chen X., Yuan L., Song C., Cheng C., Wang X., Liang X., Wang Y., Cao D., Yi H. (2021). Quantitative Tools and Applications of Time Stages Division in Human Geography Research. Econ. Geogr..

[46] Fisher W.D. (1958). On Grouping for Maximum Homogeneity. J. Am. Stat. Assoc..

[47] Sun T., Liu X., Li G. (2015). Evolution of China Spatial Economy and Regional Industrial Shift: Empirical Analysis of Changes in Economic Shares of Chinese Provinces from 1952 to 2010. Sci. Geogr. Sin..

[48] Luo H., Li B. (2021). International Structure Analysis and National Power Measurement: International Relations Network Analysis Based on Big Data. World Econ. Polit..

[49] World Bank Group (2019). Belt and Road Corridor Economics: Opportunities and Risks of Transport Corridors.

[50] Borgatti S.P. (2005). Centrality and Network Flow. Soc. Netw..

[51] Bouzzine Y.D., Lueg R. (2020). The Contagion Effect of Environmental Violations: The Case of Dieselgate in Germany. Bus. Strategy Environ..

[52] Wang Y., Song C., Sigley G., Chen X., Yuan L. (2020). Using Social Networks to Analyze the Spatiotemporal Patterns of the Rolling Stock Manufacturing Industry for Countries in the Belt and Road Initiative. ISPRS Int. J. Geo-Inf..

[53] Mugerman Y., Sade O., Shayo M. (2014). Long-Term Savings Decisions: Financial Reform, Peer Effects, and Ethnicity. J. Econ. Behav. Organ..

[54] Bramoullé Y., Habiba D., Bernard Fortin (2020). Peer effects in networks: A survey. Annu. Rev. Econ..

